# Neuroprotective roles of klotho: Molecular pathways and therapeutic implications for cognitive health in neurological and psychiatric diseases

**DOI:** 10.1113/EP093571

**Published:** 2026-04-15

**Authors:** Amir Arsalan Ghahari, Leylinaz Safaeihavadaragh, Mehrdad Nourizadeh, Shaghayegh Davari, Saba Safaei, Farshad Zare, Safa Alkayyat, Erfan Kamali Far

**Affiliations:** ^1^ Neurosciences Research Center Tabriz University of Medical Sciences Tabriz Iran; ^2^ Semmelweis University Budapest Hungary; ^3^ School of Medicine Arak University of Medical Sciences Arak Iran; ^4^ Student Research Committee, School of Medicine Tabriz University of Medical Sciences Tabriz Iran; ^5^ Department of Medical Analysis, Medical Laboratory Technique College The Islamic University Najaf Iraq; ^6^ Centre of Research Impact and Outcome Chitkara University Rajpura Punjab India

**Keywords:** Alzheimer's disease, cognitive decline, klotho, neuroprotection, pharmacological intervention, synaptic plasticity

## Abstract

Klotho, a pleiotropic protein initially identified for its role in kidney function, has garnered significant attention for its neuroprotective properties in various neurodegenerative diseases. It regulates key processes, such as oxidative stress, neuroinflammation, synaptic plasticity and myelination, all crucial for maintaining neuronal integrity and cognitive function. Preclinical studies demonstrate that klotho enhances amyloid‐β and tau clearance, stabilizes synaptic function, supports oligodendrocyte maturation and mitigates excitotoxicity. Clinical findings show that higher α‐klotho levels are associated with slower cognitive decline in Alzheimer's and Parkinson's diseases, improved outcomes in multiple sclerosis and better recovery after stroke. Notably, the protective effects of klotho appear most pronounced in individuals with high genetic or pathological risks, such as apolipoprotein E ε4 carriers in Alzheimer's disease. Despite these promising insights, the clinical application of klotho‐based therapies is hindered by variability in biomarker assays, challenges in crossing the blood–brain barrier, and the need for precision‐based interventions tailored to individual genetic profiles. Current strategies to enhance klotho activity include lifestyle changes, pharmacological agents, recombinant protein delivery and gene therapy. Although challenges remain, klotho stands as a potential therapeutic target for mitigating cognitive decline and promoting brain health across a range of neurodegenerative and cerebrovascular conditions.

## Abstract (highlights)

- **What is the topic of this review?**
  How does the anti‐ageing protein klotho support cognitive resilience across major neurological and psychiatric disorders? We integrate molecular, cellular, systems‐level and clinical data spanning Alzheimer's disease, Parkinson's disease, multiple sclerosis, amyotrophic lateral sclerosis, ischaemic stroke and mood disorders, focusing on ageing‐related vulnerability.
- **What advances does it highlight?**
  Klotho influences core nodes relevant to cognition, including redox and mitochondrial stability, neuroimmune regulation, proteostasis, synaptic function, myelin and axon integrity and neurovascular stability. Klotho has direct CNS actions, with implications that link *KL* genotype and baseline klotho levels to feasible intervention strategies, including lifestyle approaches, repurposed pharmacological inducers, recombinant protein delivery and gene‐based modulation.

## 1 INTRODUCTION

Klotho (KL) is increasingly recognized as a central modulator of biological resilience in ageing, with relevance that extends well beyond its original characterization in renal physiology. Neurodegenerative and neuropsychiatric disorders, including Alzheimer's disease (AD), Parkinson's disease (PD), multiple sclerosis (MS), amyotrophic lateral sclerosis (ALS), stroke‐related cognitive impairment and mood disorders, remain major drivers of long‐term disability and premature mortality worldwide. Despite advances in biomarkers and symptomatic therapies, no current intervention reliably halts or reverses progressive cognitive decline. Across these conditions, convergent pathobiological pressures repeatedly emerge, including oxidative stress, chronic neuroimmune activation, excitotoxicity, impaired proteostasis, synaptic dysfunction, myelin and axon injury and neurovascular disruption, which together erode neuronal integrity and destabilize large‐scale brain networks (Moos et al., 2020; Pathare & Shalia, 2019).

Klotho was first identified in 1997 as an ageing‐suppressor gene in mice and encodes a transmembrane protein that can be cleaved to yield soluble forms measurable in CSF, serum and urine. Although klotho is classically described as a coreceptor for fibroblast growth factor 23 (FGF23) in phosphate metabolism, accumulating evidence supports a broader role as a homeostatic regulator that tunes stress responses and tissue repair programmes across organ systems (Abraham et al., 2016). In the CNS, klotho has been linked to synaptic plasticity, myelination, antioxidant defences, neuroimmune set‐points and clearance pathways relevant to amyloid‐β (Aβ) and tau biology (Shaker et al., 2025; Zeldich et al., 2019). Observational studies further suggest that higher CSF or circulating α‐klotho levels are associated with better cognitive performance and slower decline, with effects that appear most evident in individuals carrying elevated genetic or pathological risk, although findings in unselected community cohorts remain less consistent (Ananya et al., 2023; Gaitan et al., 2022).

However, two limitations have constrained interpretability and translation. First, mechanistic findings are often presented as a list of downstream effects rather than as an integrated framework showing how klotho engages a limited number of convergent signalling nodes to produce coherent neuroprotective outcomes. Second, because klotho has strong peripheral actions, it remains unclear which cognitive benefits reflect direct CNS signalling in neurons, glia and the neurovascular unit versus indirect pathways, in which improved systemic homeostasis, including reduced inflammation, metabolic stress and vascular risk, secondarily shape brain immune tone and circuit resilience (Kakar et al., 2021; Zeldich et al., 2014). These gaps complicate target validation, biomarker interpretation and the design of precision trials.

In this review, we synthesize molecular, cellular, systems‐level and clinical evidence to position klotho as a convergent pro‐homeostatic factor for cognitive resilience. We organize findings across major neurological and psychiatric conditions around shared mechanistic nodes, including redox control, neuroimmune regulation, proteostasis, synaptic function, myelin and axon integrity and neurovascular stability, and we link *KL* genotype and baseline klotho levels to feasible intervention strategies spanning lifestyle approaches, repurposed pharmacological inducers, recombinant protein delivery and gene‐based modulation. Several recent reviews published in 2025 have summarized klotho biology in ageing and disease (López‐Valdés et al., 2025; Porcari et al., 2025). In contrast, the present review emphasizes a cognition‐centred mechanistic framework and a translational stratification logic designed to generate trial‐relevant hypotheses for preserving cognitive health.

## 2 MECHANISTIC FRAMEWORK: CONVERGENT PATHWAYS AND DIRECT VERSUS INDIRECT BRAIN EFFECTS OF KLOTHO

Klotho is an ageing suppressor protein with neuroprotective and cognition‐supporting actions that converge on key homeostatic nodes. First, klotho enhances redox and mitochondrial resilience. Klotho deficiency increases oxidative damage in the CNS (Hanson et al., 2021), whereas higher klotho activity upregulates redox regulators, such as thioredoxin and peroxiredoxin systems, and antioxidant enzymes, including superoxide dismutase (Zhou et al., 2018). Klotho also engages longevity pathways, including FOXO and Nrf2, and can suppress PI3K/Akt/mTOR signalling, increasing catalase and manganese superoxide dismutase (MnSOD) in glia (Orellana et al., 2023). Second, Klotho restrains neuroimmune activation. It inhibits nuclear factor kappa B (NF‐κB) signalling and reduces pro‐inflammatory cytokines, such as tumor necrosis factor alpha (TNF‐α) and interleukin‐1β (IL‐1β), in stressed neural cells (Kurosu et al., 2005). In disease models, klotho limits inflammatory amplification by inhibiting the NLRP3 inflammasome and promoting a reparative microglial phenotype that improves clearance of toxic aggregates, including Aβ. These effects reduce chronic cytokine exposure and protect vulnerable neuronal circuits (Rana et al., 2025). Third, klotho supports proteostasis by promoting autophagy and lysosomal clearance of pathogenic proteins. In an AD mouse model, cerebral Klotho overexpression inhibited Akt/mTOR signalling, activated autophagy, and promoted amyloid‐β clearance (Zeng et al., 2019). Soluble klotho can also enhance ubiquitin proteasome activity, improve turnover of misfolded proteins and reduce proteotoxic stress linked to cognitive decline (Hanson et al., 2021). Fourth, klotho strengthens synaptic plasticity. It enhances glutamatergic signalling by increasing NMDA receptor function, particularly glutamate ionotropic receptor N‐methyl‐D‐aspartate type subunit 2B (GluN2B)‐dependent signalling, which supports long‐term potentiation and memory. Peripheral soluble klotho acutely improved hippocampal plasticity and cognition through GluN2B‐dependent mechanisms (Leon et al., 2017). Klotho also preserves synaptic structure, including postsynaptic scaffolding proteins, such as postsynaptic density protein 95 (PSD‐95). Fifth, klotho promotes myelin and axonal integrity. Klotho knockout animals show impaired axonal transport and delayed oligodendrocyte maturation (Rana et al., 2025), whereas Klotho supplementation accelerates remyelination by promoting oligodendrocyte precursor differentiation and increasing myelin protein expression (Torbus‐Paluszczak et al., 2018). Sixth, klotho supports neurovascular stability. Klotho delivery reduced blood–brain barrier permeability and downregulated endothelial mediators, such as ICAM‐1, VCAM‐1 and MMP‐9, in inflammatory demyelination models (Maleki et al., 2025). Notably, peripheral klotho can improve cognition without entering the brain, consistent with vascular or perivascular signalling that relays protective cues to neural tissue (Hanson et al., 2021). Overall, klotho influences brain function through both direct and indirect routes. Direct CNS actions arise from local expression in regions such as the choroid plexus and from CNS‐targeted gene delivery, which improves plasticity and reduces neuropathology (Hanson et al., 2021; Orellana et al., 2023). Indirect systemic actions arise from endocrine α‐klotho and include effects on insulin and insulin‐like growth factor‐1 signalling and systemic inflammation that secondarily shape brain resilience (Hu et al., 2013).

## 3 KLOTHO IN ALZHEIMER'S DISEASE

AD is the most common neurodegenerative disorder and remains a leading cause of dementia worldwide. Its core pathological features include extracellular Aβ deposition, intracellular accumulation of hyperphosphorylated tau, progressive synaptic loss and gradual cognitive decline, driven by interacting processes, such as oxidative stress, neuroinflammation, impaired proteostasis and disrupted synaptic transmission (Driscoll et al., 2023, 2024). Because klotho can modulate several of these pathways at once, it is positioned as a plausible upstream modifier of AD pathogenesis and progression (Cook et al., 2023). In AD, the neuroprotective profile of klotho aligns strongly with three mechanistic nodes: proteostasis, neuroimmune modulation and synaptic stability. These correspond to its roles in facilitating amyloid and tau clearance, dampening microglial activation and maintaining NMDA receptor function and postsynaptic integrity. Evidence supports both direct CNS actions, including enhanced microglial phagocytosis and synaptic preservation, and indirect influences mediated by improved systemic redox balance and inflammatory tone (Zhao et al., 2020).

### 3.1 Evidence from human biomarker studies

Clinical studies consistently report that reduced CSF and serum α‐klotho levels are associated with higher Aβ and tau burden and poorer cognitive performance (Grøntvedt et al., 2022). Conversely, klotho variant‐single nucleotide polymorphism heterozygosity (*KL*‐VS^het^) has been linked to more favourable Aβ42 to tau ratios and slower cognitive decline, particularly in amyloid‐positive individuals (Neitzel et al., 2021). Higher CSF α‐klotho in early AD is associated with better memory scores independent of *APOE* ε4 status (Katonova et al., 2025). However, large community cohorts, such as the UK Biobank, have not reproduced a clear cognitive advantage for *KL*‐VS^het^, suggesting that klotho‐related benefits might be most evident in genetically or pathologically enriched populations rather than in generally healthy ageing groups (Chen et al., 2023).

### 3.2 Preclinical mechanistic insights

In transgenic AD mouse models, klotho overexpression reduces Aβ plaque burden by enhancing microglial phagocytosis and increasing Aβ‐degrading enzymes, such as neprilysin, while also promoting autophagic clearance (Zhao et al., 2020). At the same time, klotho limits tau pathology through inhibition of glycogen synthase kinase‐3 beta (GSK‐3β) activity, which lowers tau phosphorylation (Shibagaki et al., 2024; Wang et al., 2021). These actions preserve synaptic structure and function, including maintenance of PSD‐95 and stabilization of GluN2B‐containing NMDA receptors, leading to improved long‐term potentiation and better performance on spatial memory tasks (Zhao et al., 2020). Mechanistic findings therefore mirror human biomarker data, in which higher klotho is associated with more favourable amyloid and tau profiles and better cognition (Cook et al., 2024).

### 3.3 Genetic interactions and resilience

*KL*‐VS^het^ appears to confer resilience in individuals with high genetic risk. *APOE* ε4 carriers who also carry *KL*‐VS^het^ show lower rates of amyloid PET positivity and attenuated amyloid‐dependent tau accumulation compared with those lacking the variant. These observations support a gene–gene interaction, in which klotho modifies the impact of *APOE* ε4 and might shift the trajectory of downstream pathology (Cook et al., 2024).

### 3.4 Critical appraisal

**FIGURE 1:**
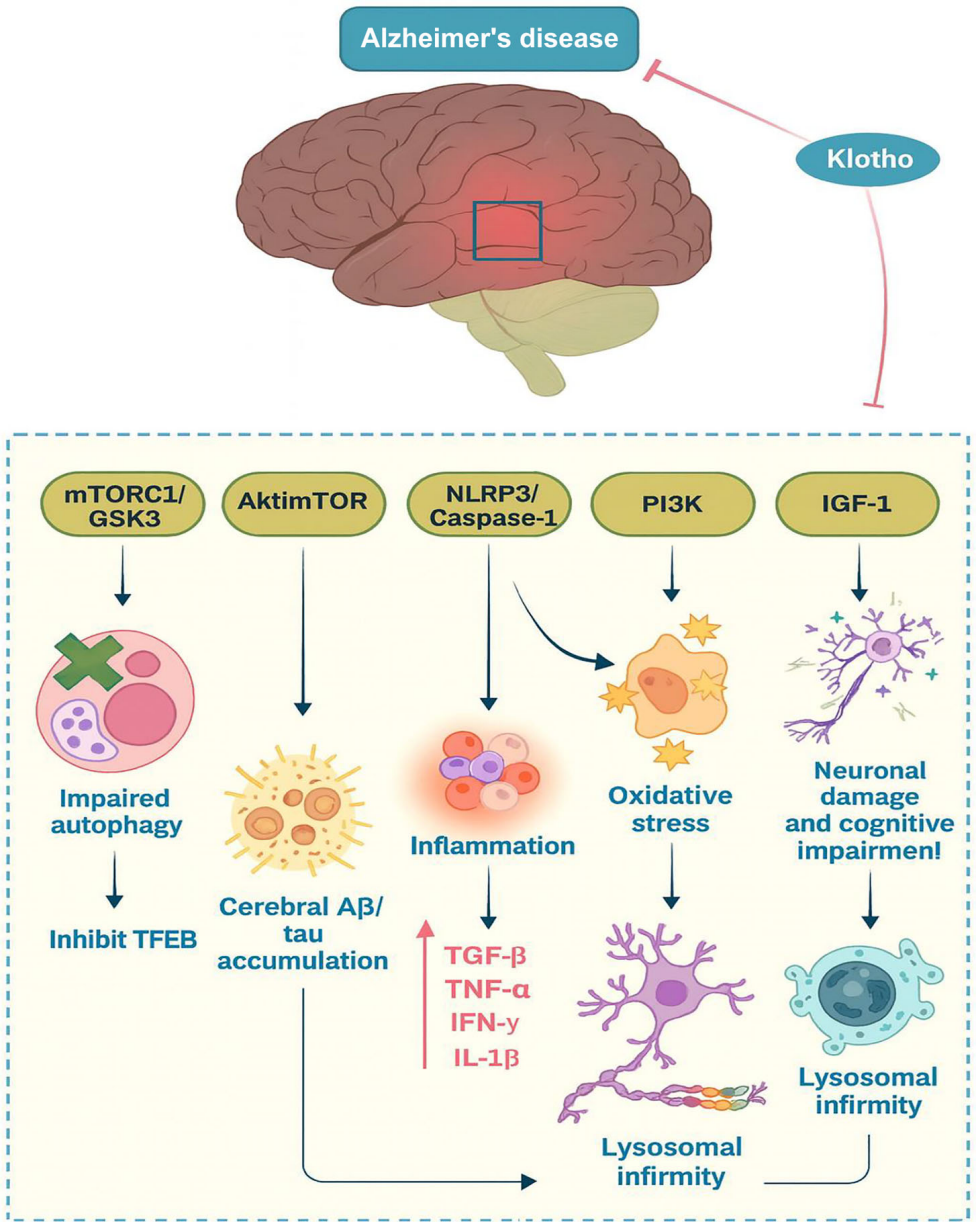
Klotho, an anti‐ageing protein, plays a protective role against the progression of Alzheimer's disease. Excessive insulin‐like growth factor‐1 (IGF‐1) stimulation in Alzheimer's disease activates the Akt/mTOR pathway, leading to autophagy disruption, inhibition of transcription factor EB (TFEB) and activation of mTORC1/GSK3. These alterations promote mitochondrial and lysosomal dysfunction, oxidative stress and the accumulation of amyloid‐β (Aβ) and tau, thereby contributing to cognitive decline and neuronal injury. Additionally, activation of the NLRP3/caspase‐1 inflammasome amplifies neuroinflammation. Klotho counteracts these processes by inhibiting PI3K/Akt/mTOR signalling, restoring autophagy and TFEB function and reducing Aβ/tau build‐up. It also suppresses mTORC1/GSK3 activity and dampens NLRP3/caspase‐1‐mediated inflammation, ultimately preserving mitochondrial function and neuronal survival. Through these mechanisms, klotho mitigates multiple pathological drivers of Alzheimer's disease and supports cognitive function.

Despite compelling convergent evidence, several limitations remain. Many human studies are cross‐sectional, with modest sample sizes, and use heterogeneous α‐klotho assays, which complicates direct comparison and causal inference. Longitudinal work with standardized measurement protocols is needed to clarify whether peripheral klotho levels reliably track CNS biology over time and to define clinically meaningful thresholds. In addition, variability in responses across genotypes underlines the importance of integrating klotho status and *KL*‐VS haplotype into precision medicine frameworks for future trials. Overall, klotho facilitates Aβ and tau clearance and preserves synaptic health, with the most pronounced effects in individuals who carry substantial genetic or pathological risk. These properties make klotho a strong candidate for targeted disease‐modifying strategies in AD (Chauhan et al., 2022; Shibagaki et al., 2024) (Figure 1).

## 4 KLOTHO IN PARKINSON'S DISEASE

PD is the second most common neurodegenerative disorder and is characterized by loss of dopaminergic neurons in the substantia nigra, α‐synuclein aggregation and both motor and non‐motor symptoms that often include cognitive decline. These features arise from oxidative stress, mitochondrial dysfunction, neuroinflammation and abnormal protein aggregation, and klotho appears to modulate several of these processes in parallel (Hosseini et al., 2024). In PD, the effects of klotho primarily converge on oxidative stress regulation, mitochondrial homeostasis and neuroimmune modulation. These mechanisms underlie its role in protecting dopaminergic neurons, enhancing autophagic clearance of α‐synuclein and reducing microglia‐driven inflammation. Evidence suggests both direct actions within the CNS and indirect benefits mediated by systemic redox balance and anti‐inflammatory signalling contribute to disease modification (Baluchnejadmojarad et al., 2017).

### 4.1 Human observational evidence

Both cross‐sectional and longitudinal studies show lower serum and CSF α‐klotho levels in PD compared with control subjects (Kakar et al., 2021; Sancesario et al., 2021; Yalcin et al., 2025). Higher baseline CSF α‐klotho predicts a later onset of cognitive impairment, especially in glucosylceramidase beta 1 (*GBA1*) mutation carriers, whereas reduced levels are linked to faster cognitive decline and worsening non‐motor symptoms, consistent with a general neuroprotective role (Zimmermann et al., 2024).

### 4.2 Preclinical mechanistic insights

In 1‐methyl‐4‐phenyl‐1,2,3,6‐tetrahydropyridine (MPTP) and 6‐hydroxydopamine PD models, klotho overexpression or recombinant protein delivery protects nigrostriatal neurons by boosting manganese MnSOD and catalase activity, stabilizing mitochondrial function, and suppressing microglial activation and cytokines, such as TNF‐α and IL‐1β. Experimental data also indicate that klotho supports calcium homeostasis and might reduce α‐synuclein aggregation through enhanced autophagy–lysosomal clearance (Hosseini et al., 2024).

### 4.3 Genetic interactions and cognitive outcomes

*KL*‐VS heterozygosity has been less studied in PD than in AD, but available evidence suggests that it might slow cognitive decline, particularly in GBA1‐associated PD, mirroring patterns seen in genetically high‐risk AD (Roeben et al., 2024). In line with this, higher circulating or CSF α‐klotho is associated with better preservation of executive function and working memory, probably reflecting its stabilizing effects on frontostriatal and hippocampal circuits (Kakar et al., 2021).

### 4.4 Translational potential

The convergence of human biomarker data and animal studies positions klotho as both a candidate prognostic biomarker and a therapeutic target in PD (Zimmermann et al., 2024). Progress towards clinical application will depend on standardized klotho assays, clearer mapping between peripheral and CNS levels, and stratified trial designs that focus on genetically enriched subgroups most likely to benefit from klotho‐based interventions (Shen et al., 2025).

## 5 KLOTHO IN MULTIPLE SCLEROSIS AND DEMYELINATING DISORDERS

MS is a chronic inflammatory demyelinating disease of the CNS, in which immune‐mediated myelin loss leads to axonal injury and progressive neurodegeneration. Focal lesions, together with diffuse white and grey matter damage, drive accumulating disability and cognitive decline. Recent work suggests that klotho helps to regulate oligodendrocyte biology, myelination and neuroimmune balance, which places it as a potential therapeutic target in MS (Baranowska & Kochanowski, 2020). In MS, the actions of klotho are most prominently aligned with myelin–axon integrity and neuroimmune regulation. By promoting oligodendrocyte progenitor cell maturation and reducing microglial activation, klotho supports remyelination and dampens inflammatory injury. These effects involve both direct mechanisms within the CNS and systemic influences that modulate immune tone and oxidative stress (Maleki et al., 2025; Zeldich et al., 2015).

### 5.1 Human biomarker evidence

Clinical studies consistently report lower serum and CSF α‐klotho levels in people with MS compared with age‐matched control subjects, with the most pronounced reductions in progressive forms (Birdi et al., 2024). Decreased α‐klotho is correlated with higher Expanded Disability Status Scale (EDSS) scores, greater lesion load, more brain atrophy on MRI, and reduced fractional anisotropy on diffusion tensor imaging, indicating disrupted white matter microstructure. Together, these findings suggest that klotho is linked to both the inflammatory demyelinating and neurodegenerative components of MS (Kuriyama et al., 2018).

### 5.2 Mechanistic insights from experimental models

In demyelination models, such as cuprizone exposure and experimental autoimmune encephalomyelitis, klotho overexpression or supplementation accelerates remyelination. It promotes the differentiation of oligodendrocyte progenitor cells into mature myelinating oligodendrocytes by activating extracellular signal‐regulated kinases 1 and 2 (ERK1/2) and PI3K/Akt signalling, which support cell survival and myelin protein synthesis. At the same time, klotho suppresses Wnt/β‐catenin signalling, a pathway that keeps progenitors in an undifferentiated state, and dampens microglial activation and pro‐inflammatory cytokine production, thereby reducing inflammatory damage (Chen et al., 2020).

### 5.3 Cognitive relevance in MS

Cognitive impairment affects ∼40%–65% of individuals with MS, typically involving memory, attention and processing speed. Lower α‐klotho levels have been associated with poorer performance on standardized neuropsychological tests (Birdi et al., 2024). This pattern implies that the benefits of klotho extend beyond myelin preservation and also involve the protection of neurons and synapses that support cognitive networks (Castner et al., 2023).

### 5.4 Translational considerations

Because klotho can both modulate immune responses and enhance remyelination, it represents an attractive adjunctive target alongside established MS therapies (Birdi et al., 2024). Translation to the clinic will require approaches that achieve effective klotho levels within the CNS despite blood–brain barrier constraints. Early studies of intranasal delivery and small‐molecule inducers are beginning to address this challenge. Overall, klotho in MS appears to act as an immunomodulator and remyelination enhancer that can influence disease progression and cognition by supporting oligodendrocyte maturation and preserving white matter integrity (Castner et al., 2023).

## 6 KLOTHO IN AMYOTROPHIC LATERAL SCLEROSIS AND MOTOR NEURON DISEASES

ALS is a rapidly progressive and fatal neurodegenerative disease marked by degeneration of upper and lower motor neurons, leading to paralysis and respiratory failure. Its pathophysiology involves oxidative stress, mitochondrial dysfunction, glutamate‐driven excitotoxicity, protein aggregation and chronic neuroinflammation. Cognitive and behavioural changes are increasingly recognized, placing ALS within a broader neurodegenerative spectrum. Clinical and experimental data suggest that klotho might modify disease course by enhancing antioxidant defenses, limiting neuroinflammation and preserving synaptic integrity (Chen et al., 2020). In ALS, the neuroprotective mechanisms of klotho span oxidative stress regulation, neuroimmune restraint, synaptic stabilization and support for axon–glial integrity. These actions reflect its capacity to reduce glial activation, enhance antioxidant enzyme expression and preserve neuromuscular connectivity. Both direct CNS effects and peripheral modulation of inflammation and metabolic stress are likely to contribute to its disease‐modifying potential (Zeldich et al., 2019).

### 6.1 Human biomarker evidence

Patients with ALS show significantly reduced serum α‐klotho compared with healthy control subjects. Lower levels are associated with faster decline on the ALS Functional Rating Scale Revised (ALSFRS‐R), shorter survival and more rapid loss of respiratory function. Although large prospective cohorts are still limited, current evidence supports α‐klotho as a potential prognostic biomarker that warrants validation in longitudinal studies (Chen et al., 2020).

### 6.2 Mechanistic insights from experimental models

In *SOD1* ^G93A^ ALS mouse models, klotho overexpression delays disease onset, slows motor deterioration and extends survival, with more pronounced effects in females (Orellana et al., 2023). Mechanistically, klotho reduces oxidative stress by upregulating MnSOD and catalase in motor neurons; it inhibits NF‐κB signalling and thereby restricts microglial and astrocytic activation, and it preserves neuromuscular junctions while limiting distal axonal degeneration. It might also improve the function of myelinating Schwann cells and thereby support peripheral nerve conduction (Zeldich et al., 2019).

### 6.3 Functional and cognitive implications

Although ALS is classically defined by motor dysfunction, many patients display frontotemporal behavioural and cognitive changes. By reducing oxidative stress and supporting synaptic stability, klotho could influence both motor and non‐motor manifestations. Direct evidence for cognitive benefits in ALS is still sparse; however, parallels with other klotho‐responsive neurodegenerative disorders justify further investigation into its broader neurocognitive impact (Zeldich et al., 2019).

### 6.4 Translational potential

The survival benefits observed in animal models position klotho as a candidate disease‐modifying approach in ALS. Experimental strategies include gene therapy, recombinant protein administration and pharmacological induction of endogenous klotho, all of which show preliminary promise (Hosseini et al., 2024). Stratifying patients according to baseline klotho levels might help to identify subgroups most likely to benefit in future trials (Paquette et al., 2023). Overall, klotho in ALS appears to exert a consistent neuroprotective profile that goes beyond oxidative stress control and includes dampening of neuroinflammation, preservation of synapses and stabilization of neuromuscular connections, supporting its development as an adjunctive therapy for motor neuron diseases (Zeldich et al., 2019).

## 7 KLOTHO IN STROKE, ISCHAEMIA AND POSTISCHAEMIC COGNITIVE DECLINE

Stroke is a major cause of long‐term disability and cognitive impairment, most commonly attributable to ischaemic events. The resulting brain injury triggers oxidative stress, excitotoxicity, neuroinflammation and blood–brain barrier disruption that can extend beyond the acute phase and drive ongoing neurodegeneration. Recent data suggest that klotho has protective effects both during acute ischaemic injury and in the prevention or attenuation of postischaemic cognitive decline (Jin et al., 2021). In ischaemic stroke and its cognitive sequelae, the actions of klotho converge on redox regulation, neurovascular stability and neuroimmune modulation. It reduces oxidative damage and inflammation during acute injury, supports blood–brain barrier integrity and enhances post‐stroke recovery via pathways such as the irisin–klotho axis. These effects reflect both direct CNS actions and peripheral mechanisms involving muscle‐derived signals and systemic anti‐inflammatory tone (Jin et al., 2021).

### 7.1 Acute phase changes in klotho levels

Clinical studies show that CSF α‐klotho concentrations fall markedly within hours to days after ischaemic stroke. Lower early post‐stroke levels are correlated with higher National Institutes of Health Stroke Scale (NIHSS) scores, larger infarct volumes on neuroimaging, and worse 90 day functional outcomes on the modified Rankin scale. These findings imply that ischaemia rapidly depletes endogenous klotho signalling, which might amplify secondary injury and limit neurological recovery (Zhu et al., 2023).

### 7.2 Mechanistic insights from experimental models

In middle cerebral artery occlusion models, klotho overexpression or recombinant protein delivery confers robust neuroprotection. These interventions reduce reactive oxygen species and lipid peroxidation, preserve mitochondrial membrane potential and ATP production and inhibit NF‐κB activation, with downstream suppression of TNF‐α and IL‐1β. Klotho also helps to maintain blood–brain barrier integrity by preserving tight junction proteins, thereby limiting infarct expansion and supporting better functional recovery after ischaemia (Zhu et al., 2023).

### 7.3 The irisin–klotho axis

Work by Jin et al. indicates that irisin, a myokine released during physical activity, improves post‐stroke cognition through klotho‐dependent pathways. In mice with middle cerebral artery occlusion, both irisin administration and pre‐stroke swimming increased hippocampal klotho expression, restored antioxidant enzyme activity and enhanced memory performance. These effects were absent in klotho‐deficient mice, supporting a causal role for klotho in irisin‐mediated neuroprotection after ischaemic injury (Jin et al., 2021).

### 7.4 Cognitive sequelae and klotho

Up to half of stroke survivors develop post‐stroke cognitive impairment within the first year. Higher post‐stroke α‐klotho levels have been associated with better memory and executive function, suggesting that klotho might facilitate neuronal repair and reorganization of functional networks that underlie cognitive recovery (Jin et al., 2021; Liu et al., 2024).

### 7.5 Translational implications

Enhancing klotho activity after ischaemic stroke could reduce long‐term disability and dementia risk. Potential strategies include exercise‐mimetic agents targeting the irisin–klotho axis, recombinant klotho administration and pharmacological approaches that boost endogenous expression. Key uncertainties remain regarding the optimal therapeutic window, dosing and route of delivery (Jin et al., 2021) (Table 1).

**TABLE 1:** Integrated comparative summary of klotho in major neurodegenerative, demyelinating and cerebrovascular disorders.

Disorder	Human evidence (α‐klotho)	Core mechanistic nodes (cognition‐relevant)	Key modifiers	Cognitive/clinical signal	Translational angle	Reference
AD	Lower CSF/serum levels are associated with higher Aβ/tau burden and worse cognition	Proteostasis (Aβ clearance, autophagy), synaptic stability (GluN2B, PSD‐95), ↓GSK‐3β and tau phosphorylation, microglial phagocytosis	*KL‐VS* ^het^; stronger effects in *APOE* ε4 and amyloid‐positive groups	Slower cognitive decline in high‐risk strata	Recombinant *KL*, CNS gene delivery, lifestyle, renin–angiotensin–aldosterone system blockade, vitamin D analogues	Wu et al. (2023)
PD	Lower CSF/serum levels are associated with faster cognitive decline and non‐motor severity	Redox/mitochondria (↑MnSOD, catalase), ↓NF‐κB‐driven neuroinflammation, proteostasis (autophagy–lysosome, ↓α‐synuclein aggregation), Ca^2+^ homeostasis	Potential enrichment in *GBA1*‐associated PD; limited *KL*‐VS data	Delayed onset of cognitive impairment; better executive function	Recombinant KL, mTOR‐targeting strategies, antioxidants, genotype‐stratified trials	Kakar et al. (2021)
MS	Lower levels, most pronounced in progressive MS; associated with disability and markers of neurodegeneration	Myelin–axon integrity (OPC maturation), ↓Wnt/β‐catenin, immune tone and microglial activation, neurovascular stabilization	No established *KL* genotype signal; inflammation burden likely modulates	Lower levels link to poorer processing speed and memory	Intranasal KL, small‐molecule inducers, adjunct to DMTs (immunotherapies)	Risi et al. (2025)
ALS	Lower serum levels are associated with faster functional decline and reduced survival	Redox defence, ↓NF‐κB glial activation, NMJ preservation, axon–glia support	SOD1^G93A^ responsiveness; possible sex effects	Slower motor deterioration; cognitive impact plausible but less defined	Gene therapy, recombinant KL, pharmacological induction; baseline‐level stratification	Zeldich et al. (2019)
Stroke	Early post‐stroke decline in CSF; lower levels are associated with severity and worse outcome	Neurovascular stability (BBB), redox/mitochondria, ↓NF‐κB inflammation; irisin–KL axis in recovery	Irisin–KL pathway (exercise‐linked)	Better recovery and post‐stroke cognition with higher KL	Rehab/exercise, irisin mimetics, recombinant KL; time‐window optimization	Scazzone et al. (2020)

Abbreviations: Aβ, amyloid‐β; AD, Alzheimer’s disease; ALS, amyotrophic lateral sclerosis; APOE, apolipoprotein E; BBB, blood‐brain barrier; Ca²⁺, calcium; CNS, central nervous system; CSF, cerebrospinal fluid; DMTs, disease‐modifying therapies; GBA1, glucosylceramidase beta 1; GluN2B, glutamate ionotropic receptor N‐methyl‐D‐aspartate type subunit 2B; GSK‐3β, glycogen synthase kinase 3 beta; KL, Klotho; KL‐VS, Klotho‐VS haplotype; KL‐VS^het^, heterozygous KL‐VS haplotype; MnSOD, manganese superoxide dismutase; mTOR, mechanistic target of rapamycin; MS, multiple sclerosis; NF‐κB, nuclear factor kappa B; NMJ, neuromuscular junction; OPC, oligodendrocyte precursor cell; PD, Parkinson’s disease; PSD‐95, postsynaptic density protein 95; RAAS, renin‐angiotensin‐aldosterone system; α‐syn, alpha‐synuclein; SOD1^G93A^, superoxide dismutase 1 G93A mutant

## 8 THERAPEUTIC STRATEGIES FOR AUGMENTING KLOTHO

Given the extensive neuroprotective and systemic effects of klotho, several complementary strategies have been proposed to enhance its expression or function. These interventions operate across both direct and indirect pathways, targeting key mechanistic nodes, such as oxidative stress, inflammation, proteostasis and synaptic function. Lifestyle modification and repurposed pharmacological agents primarily influence peripheral physiology to support brain resilience, whereas advanced biotechnological approaches, such as gene therapy and recombinant protein delivery, aim to restore klotho activity more directly within the CNS (Abraham & Li, 2022; Moos et al., 2020).

First, lifestyle interventions might modulate circulating klotho. Aerobic and resistance exercise are associated with higher klotho levels, possibly through reduced systemic inflammation and improved metabolic signalling, including myokines, such as irisin (Corrêa et al., 2022). Mediterranean and Dietary Approaches to Stop Hypertension (DASH) style dietary patterns have also been linked to higher serum klotho, potentially by attenuating oxidative stress and low‐grade inflammation (Liu & Chen, 2023). Caloric restriction increases klotho expression in animal models, although human evidence remains limited (Shafie et al., 2020). Beyond lifestyle, several commonly used drugs might act as klotho inducers. Renin–angiotensin–aldosterone system blockers can increase renal klotho transcription by suppressing angiotensin II‐related NF‐κB activation. Statins might enhance klotho expression through endothelial and anti‐inflammatory effects (Prud'homme et al., 2022). Vitamin D analogues, such as calcitriol, can stimulate *KL* transcription, and mTOR inhibitors, such as rapamycin, might increase klotho in parallel with longevity‐related pathways (Buchanan et al., 2020).

Finally, targeted augmentation strategies include gene and protein‐based approaches. Adeno‐associated virus (AAV)‐mediated *KL* delivery can drive sustained expression in preclinical models, although safety and tissue specificity remain key barriers (Prud'homme et al., 2022). Recombinant α‐klotho has improved cognition and reduced neurodegeneration in animal studies, and delivery optimization approaches, such as PEGylation and nanoparticle formulations, are under investigation (Gajbhiye et al., 2020). Epigenetic and transcriptional activation strategies, including promoter demethylation and engineered transcription factors, offer cell‐selective upregulation but remain early stage (Jain et al., 2024).

## 9 CLINICAL IMPLICATIONS

Clinical and translational evidence increasingly supports klotho as both a biomarker and a therapeutic target in neurodegenerative and cognitive disorders. Its involvement in key mechanistic pathways, including oxidative stress, neuroinflammation, proteostasis and synaptic integrity, positions it as a useful indicator of vulnerability and progression (Elza et al., 2025). Lower CSF or serum α‐klotho levels have been linked to faster cognitive decline in diseases such as AD, PD and MS, with the strongest associations being seen in individuals with elevated genetic or pathological risk, including *APOE* ε4 carriers with amyloid positivity and PD patients with *GBA1* mutations (Raber et al., 2025). Serial assessment of klotho, especially when interpreted alongside *KL*‐VS haplotype status and disease‐specific markers, such as amyloid to tau ratios, α‐synuclein and neurofilament light chain, might refine risk stratification and improve monitoring of disease progression and treatment response (Gao et al., 2025).

Therapeutically, klotho can be targeted at several levels. Lifestyle‐based approaches, such as structured aerobic exercise and Mediterranean or DASH‐style diets, and possibly caloric restriction, have been associated with higher klotho levels, particularly in older adults and those with metabolic comorbidities (Yossef et al., 2020). Repurposed agents, including renin–angiotensin–aldosterone system inhibitors, statins and vitamin D analogues, provide pragmatic pharmacological means to increase endogenous klotho modestly, whereas recombinant protein delivery and gene‐based strategies show robust effects in preclinical models but are still constrained by issues of brain penetration, long‐term safety and regulation of expression (Zhang & Zhang, 2023). At the same time, translation into routine practice is limited by heterogeneous assay methods, the absence of standardized reference ranges and an incomplete understanding of how serum and CSF klotho relate to CNS biology (Kundu et al., 2022). Moving klotho towards clinical use will therefore require harmonized measurement protocols and a precision medicine framework that incorporates genetics, comorbidities and baseline klotho status when designing and interpreting klotho‐focused interventions (Zhang & Zhang, 2023).

## 10 DISCUSSION

Over the past two decades, klotho has shifted from a kidney‐centred anti‐ageing factor to a multisystem regulator with substantial relevance for brain health. Across AD, PD, MS, ALS, stroke and selected psychiatric conditions, evidence supports a convergent model, in which klotho promotes cognitive resilience by modulating a limited set of interconnected nodes, including oxidative stress control, neuroimmune regulation, proteostasis, synaptic function, myelin and axon integrity and neurovascular stability (Hanson et al., 2021; López‐Valdés et al., 2025). Mechanistically, klotho enhances antioxidant defences, restrains NF‐κB‐mediated inflammatory signalling in glia, supports synaptic plasticity and oligodendrocyte maturation and facilitates autophagy‐related clearance of pathogenic proteins, such as amyloid and tau (Hanson et al., 2021). These benefits are likely to reflect both direct CNS actions within neurons, glia and the neurovascular unit and indirect periphery‐to‐brain influences via improved systemic inflammation, mitochondrial efficiency, vascular integrity and metabolic homeostasis (Wang et al., 2026).

Human studies also indicate that klotho‐related cognitive advantages are not uniformly distributed. Stronger associations are typically reported in individuals with higher genetic or pathological burden, including *APOE* ε4 carriers with amyloid positivity, PD patients with *GBA1* mutations, and progressive MS, supporting a threshold‐based interpretation, in which klotho becomes most relevant when cumulative stress exceeds intrinsic neural buffering capacity (Driscoll et al., 2023). This non‐linearity underscores the importance of biomarker‐driven stratification and precision‐oriented intervention strategies (Linghui et al., 2023).

Therapeutically, klotho can be targeted through lifestyle induction, pharmacological upregulation, recombinant protein delivery, gene‐based approaches and epigenetic modulation (Chen & Zhu, 2025; Hanson et al., 2021). However, translation remains constrained by brain delivery barriers, long‐term safety uncertainty and inter‐individual variability in klotho expression and responsiveness (Kakar et al., 2021). Indirect strategies, such as engaging the irisin–klotho axis through physical activity or mimetics, might provide a scalable route to augment klotho‐related signalling (Kanbay et al., 2023). Progress will require harmonized assay platforms, longitudinal studies across diverse populations, a clearer definition of functional klotho signalling in the human brain, and trial designs integrating genotype, comorbidity burden and baseline klotho status (Kundu et al., 2022). If these challenges are addressed, klotho might serve as a unifying target to preserve cognition by acting on shared neurodegenerative mechanisms rather than isolated disease‐specific pathways (Hanson et al., 2021).

## 11 CONCLUSION

Klotho has emerged as a unifying pro‐homeostatic factor linking brain ageing, systemic physiology and neurodegeneration. Across AD, PD, MS, ALS and ischaemic stroke, evidence suggests that higher klotho activity supports cognitive resilience by converging on shared mechanistic nodes, including redox and mitochondrial stability, neuroimmune regulation, proteostasis, synaptic function and myelin and neurovascular integrity. Human data also support a threshold‐based model, in which klotho‐related benefits are most detectable in individuals with higher genetic or pathological burden, such as *APOE* ε4 carriers with amyloid positivity and PD patients with *GBA1* mutations, implying that stratified trial designs are essential. Clinical translation remains limited by heterogeneous assays, incomplete mapping of peripheral and central pools, and gaps in cell type‐specific signalling, particularly in the human brain. Therapeutic opportunities span lifestyle and pharmacological induction, in addition to recombinant and gene‐based strategies, with activity‐dependent pathways, such as irisin‐related signalling, offering physiologically aligned routes for augmentation. If these challenges are addressed, klotho might enable interventions that target shared mechanisms of brain ageing rather than single disease entities.

## AUTHOR CONTRIBUTIONS

Supervision, conceptualization, writing—original draft and writing—review and editing: Amir Arsalan Ghahari, Leylinaz Safaeihavadaragh and Mehrdad Nourizadeh Conceptualization, writing—original draft and writing review—editing: Shaghayegh Davari, Saba Safaei, Farshad Zare, Safa Alkayyat and Erfan Kamali Far All authors approved the final version of the manuscript and agree to be accountable for all aspects of the work in ensuring that questions related to the accuracy or integrity of any part of the work are appropriately investigated and resolved. All persons designated as authors qualify for authorship, and all those who qualify for authorship are listed.

## CONFLICT OF INTEREST

None declared.

## FUNDING INFORMATION

None.

## Data Availability

Data sharing is not applicable to this article because no datasets were generated or analysed during the study.
